# Scarlett Letter: A study based on experience of stigma by COVID-19 patients in quarantine

**DOI:** 10.12669/pjms.36.7.3606

**Published:** 2020

**Authors:** Nazish Imran, Hadia Afzal, Irum Aamer, Ali Hashmi, Bilquis Shabbir, Aftab Asif, Saeed Farooq

**Affiliations:** 1 Nazish Imran, MBBS; FRCPsych (London); MRCPsych (London); MHPE. Associate Professor, Department of Child and Family Psychiatry, King Edward Medical University/Mayo Hospital, Lahore, Pakistan; 2 Hadia Afzal, MBBS. Postgraduate Resident, Academic Department of Psychiatry and Behavioral Sciences, King Edward Medical University/Mayo Hospital, Lahore, Pakistan; 3 Irum Aamer, MBBS; FCPS. Senior Registrar, Academic Department of Psychiatry and Behavioral Sciences, King Edward Medical University/Mayo Hospital, Lahore, Pakistan; 4 Ali Madeeh Hashmi, MBBS, MD (USA), DABPN (USA), FAPA (USA), Associate Professor, Academic Department of Psychiatry and Behavioral Sciences, King Edward Medical University/Mayo Hospital, Lahore, Pakistan; 5 Bilquis Shabbir, MBBS; FCPS (Pak), Chairperson and Professor, Department of Medicine, King Edward Medical University/Mayo Hospital, Lahore, Pakistan; 6 Aftab Asif, MBBS.MRCPsych. Chairman and Professor, Academic Department of Psychiatry and Behavioral Sciences, King Edward Medical University/Mayo Hospital, Lahore, Pakistan; 7 Saeed Farooq. MBBS; FCPS (Psych); MCPS (Psych), PhD. Professor of Psychiatry and Public Mental Health, School of Primary, Community and Social Care, Keele University and Honorary Consultant Psychiatrist, Midlands Partnership NHS Foundation Trust, Staffordshire, UK

**Keywords:** Coronavirus, COVID-19, Discrimination, Stigma, Pakistan, global efforts

## Abstract

**Background and Objectives:**

Stigma around COVID-19 is a major barrier in global efforts to control the COVID 19 pandemic. Limited data is available regarding stigma faced by COVID-19 patients in low- and middle-income countries (LMIC). The aim of the current study was to explore the stigma experienced by hospitalized patients with COVID-19 illness in Lahore, Pakistan.

**Methods:**

Following Institutional Review Board approval and informed consent, patients were assessed using modified HIV short form stigma scale and open-ended questions. Questions focused on experiences, feelings, and opinions as to how patients feel and how they were treated prior to and during the hospitalization. Data analysis for quantitative data was performed using SPSS-20, while qualitative responses were interpreted by content analysis method.

**Results:**

One hundred and fourteen patients were interviewed (Mean age 38.8 years + 15.3) with 53.5% being males. Widespread experience of stigma was reported by patients particularly for concerns about public attitudes (7.43 + 1.43) & disclosure (6.89 + 1.45). Main themes which emerged from the qualitative responses were social stigma and rejection, humiliating behaviour of others, breach of confidentiality, loss of trust/ respect, and impact of COVID-19 diagnosis on their business.

**Conclusions:**

Existence of significant stigma among COVID-19 patients isolated in a tertiary care hospital in a LMIC highlights the need for culturally sensitive strategies to address it.

## INTRODUCTION

Stigma – traditionally a Greek word, denoting a permanent mark, has been used in recent decades to describe the process of negative discrimination against people with certain physical, behavioural, or social attributes.^1^ It carries the notion of a ‘spoilt identity’ and gives the bearer a feeling of “shame, guilt and disgrace”^2^

Infectious diseases like Acquired Immune Deficiency Syndrome (AIDS), Tuberculosis, and Severe Acute Respiratory Syndrome (SARS). have a well-established history of carrying stigma.^3^ While the current COVID-19 pandemic is causing massive global disruption, increasing instances of stigma and discrimination against certain communities and population groups afflicted by COVID-19 are being reported.^4^ A 10-fold increase in stigmatizing posts using the word “China virus” instead of COVID-19 on Twitter was noticed in all 50 states of USA following the March 16, 2020 use of this term by the United States President.^5^ A field hospital in Dhaka, Bangladesh was destroyed by locals due to fear of contamination and spread of the Corona Virus.^6^ Similarly, communities in Pakistan have refused to bury the victims of this deadly virus in local graveyards.^7^

Stigma associated with COVID-19 is identified as a significant barrier for global efforts to control the pandemic. The World Health Organization (WHO) Director-General emphasized that “This is the time for facts, not fear. This is the time for science, not rumours. This is the time for solidarity, not stigma”.^8^ It is noteworthy that in low and middle income countries like Pakistan, stigma and fear of being labelled a ‘mortal danger to others’ are more likely to interfere with delay in testing, denying early clinical symptoms and seeking timely medical interventions, thus increasing the disease burden.^9^ Quarantined individuals are more likely to report stigmatization and social rejection.^10^ Literature review suggests that stigmatization mostly occurs against marginalized groups and communities.^10^ In the COVID-19 pandemic Asians, the elderly, immune-compromised, and health care professionals are at risk for stigmatization. The increased stigma associated with COVID-19 may further exacerbate the psychological impact.

It is therefore important to understand the nature and degree of stigma associated with COVID 19. The specific objectives of current study were to explore stigmatization faced by confirmed and suspected COVID-19 patients hospitalized in a tertiary care setting in Lahore, Pakistan. We also aimed to understand how stigma affects various domains of the life of a COVID-19 patient, in order to assist healthcare professionals in measures to counter stigma and manage this deadly pandemic effectively.

## METHODS

Our study involved video-assisted telephone interviews with confirmed or suspected COVID-19 patients admitted in COVID-19 units of Mayo Hospital, Lahore, Pakistan from 25^th^ March to 30^th^ April 2020. Mayo Hospital was designated as the main referral centre for the care & management of COVID-19 patients during the early phase of the outbreak in the city of Lahore. Patients had suspected COVID -19 clinically as diagnosed by treating physicians or had positive rRT-PCR test for COVID-19. Telephonic video interviews were conducted due to limited personal protective equipment availability in the hospital during the early phase of the outbreak and to protect healthcare workers from unnecessary exposure to infection. Interview duration was between 45 minutes to an hour with follow up interviews required in a few cases.

Patients aged >18 years, who were medically stable and willing to cooperate in the study were included. Patients with altered mental state and medical issues making communication difficult were excluded from the study. The study was approved by the the Institutional Review Board of King Edward Medical University. (Ref. 251/RC/KEMU) All potentially eligible participants were briefed about the study and confidentiality and informed consent was taken. They were assured that the information they are disclosing would be used only for research purposes.

Assessment measures included demographic information Form and modified 12 item short version of HIV stigma scale.^11^ Demographic information collected included age, gender, containment procedures (isolation, or quarantine), and previous medical or psychiatric history (Table-I). HIV stigma scale has been used to study stigma related to other infectious diseases. Modified Short version of HIV stigma scale is divided into 4 sub-scales (with 3 items each) intended to measure personalized stigma, disclosure concerns, concerns about public attitudes & negative self-image.^11^ Concerns about public attitudes and disclosure include a measure of the discernment of societal stereotyping, marginalization and discrimination in terms of trust issues. Personalized stigma and negative self-image explore feelings about embarrassment, feeling different and incompatible from others and having a sense of putting others at unease by one’s presence. Each point is scored on a Likert scale of 1-4. Higher scores indicate greater stigmatization. Modified short version of HIV stigma scale was translated into the Urdu language by translation into Urdu and back translation into English to ensure reliability in the local setting prior to the study. Cronbach’s alpha for subscales ranged from 0.7-0.8. (Table-II).

**Table-I:** Clinical and Sociodemographic Characteristics of Respondents admitted in hospital related to COVID-19 Illness.

Characteristics	No	%
Number of days since Admission to Hospital: Mean (S.D)	7.85(4.1)	
Age: Mean (S.D)	38.8(15.3)	
*Gender*		
Men	61	53.5
Women	53	46.5
*Marital Status*		
Unmarried Married	27 85	23.7 74.6
Separated/ Divorced	2	1.8
*Occupation*		
unemployed	2	1.7
House wife	24	21.0
Student	19	16.6
Unskilled Worker	24	21.0
Skilled Worker	46	40.3
*Education status*		
Illiterate	6	5.3
Upto Grade 5	19	16.7
Upto Matric (year 10 secondary school)	48	42.1
Graduation	30	26.3
Professional degree	11	9.6
*Area of residence*		
Urban	107	93.9
Rural	7	6.1
*Travel History:*		
Yes	13	11.4
No	101	88.6
*Confirmed history of contact with Covid-19 Patient:*		
Yes	65	57.0
No	49	43.0
*Any other family member also suffering from COVID-19*		
Yes	67	58.8
No	47	41.2

**Table-II:** Descriptive statistics for subscales and items in the Modified short-form version of the Stigma Scale.

	Mean subscale score^b^ (SD)	Mean item score (SD)^c^
**Personalized stigma** (Cronbach’s alpha=0.73)	6.82(1.28)	
Some people avoid touching me once they know I have COVID-19.		2.50(0.59)
People I care about stopped calling after learning I have COVID-19.		2.14(0.47)
I have lost friends by telling them I have COVID-19.		2.18(0.50)
**Disclosure concerns** (Cronbach’s alpha=0.87)	6.89(1.45)	
Telling someone I have COVID-19 is risky.		2.34(0.57)
I work hard to keep my COVID-19 a secret.		2.29(0.54)
I am very careful who I tell that I have COVID-19.		2.25(0.51)
**Concerns about public attitudes** (Cronbach’s alpha=0.77)	7.43(1.43)	
People with COVID-19 are treated like outcasts.		2.52(0.59)
Most people believe a person who has COVID-19 is dirty.		2.37(0.56)
Most people are uncomfortable around someone with COVID-19.		2.54(0.56)
**Negative self-image** (Cronbach’s alpha=0.82)	6.72(1.34)	
I feel guilty because I have COVID-19.		2.22(0.49)
People’s attitudes about COVID-19 make me feel worse about myself.		2.30(0.54)
I feel I’m not as good a person as others because I have COVID-19.		2.20(0.51)

b) Possible score 3–12 on each scale; higher scores reflect a higher level of perceived COVID-19-related stigma,

c) Possible score for each item 1–4; higher scores reflect a higher level of perceived COVI-19-related stigma. SD: Standard deviation

The research team added three questions to capture the patients experience of being diagnosed and receiving treatment for COVID-19. These questions were developed in consultation with the physicians who treated these patients. The questions were open ended and covered following areas: Patients experience and feelings when they realized that they were infected or suspected of being infected with Coronavirus and had to be isolated/quarantined in hospital, their opinion of how they were and are being treated by everyone including healthcare workers during hospitalization and their main concerns during hospital stay. The response to these questions were recorded in verbatim.

Data analysis for quantitative data was performed using SPSS version 20.0 (SPSS Inc., Chicago, IL, USA). Descriptive statistics were calculated for demographic data and subscale scores of the Stigma scale. An independent sample t test was used to test for differences in mean Stigma subscale scores between groups [Gender, duration of quarantine of one week or more). Statistical significance was set at *P* <0.05. The analysis of open-ended questions aimed to identify key characteristics of experiences of discrimination and stigma. Regarding qualitative responses to open ended questions, patients’ responses were coded and interpreted by Content Analysis Method. Two researchers independently reviewed and compared the answers several times to ensure the accuracy and recode them by agreement.

## RESULTS

Overall, 114 patients [61(53.5%) male and 53(46.5%)] participated. Among these, 102 (89.5%) were confirmed cases based on rRT-PCR test and 12 (10.6 %) were suspected COVID-19 cases. The mean age was 38.8±15.3 (range, 16–67) years (Table-I). Mean duration of hospital admission was 7.9±4.1 days. Additional demographic information is given in Table-I.

Widespread experience of stigma was reported by patients on different subscales of our assessment tool. Descriptive statistics for the scale are presented in Table-II at the item level and subscale level. Highest score were recorded on the scale of concerns about public attitudes (7.43 + 1.43) and on the questions that read “Most people are uncomfortable around someone with COVID-19” and “People with COVID-19 are treated like outcasts”.

Regarding personalized stigma, majority rated that “Some people avoid touching me once they know I have COVID-19” although this may not be considered as stigmatizing behaviour in context of COVID-19. Furthermore, it came out to be a common perception that “telling someone I have COVID-19 is risky” (Table-II). Males experienced more personalized stigma (*p-* value <.05). Table-III.

**Table-III:** Gender Differences in stigma subscales.

Variables	Male		Females		P-Value

	M	SD	M	SD	
Personalized stigma	7.05	1.39	6.55	1.08	.036*
Disclosure concerns	7.11	1.60	6.62	1.22	.072
Concerns about public attitudes	7.41	1.46	7.45	1.40	.874
Negative self-image	6.82	1.46	6.60	1.18	.393

* *p-* value <.05

Qualitative data from interviews with patients provides insight into the stigma experienced by hospitalized patients related to COVID-19. Main themes which emerged from responses were social stigma and rejection, humiliating and sarcastic behavior of others, breach of confidentiality and loss of trust and respect, and impact of COVID-19 diagnosis on their business.

### Social stigma and rejection

The research participants claimed that their family’s social relationship have been affected and their families are under pressure from society. For example,

“I have two kids at home. Ever since I am here, they are facing a lot negative treatment back home. Parents in the neighbourhood are not letting their kids play with my sons, even though they tested negative.”(P39, 38 years old female)

“As soon as the street in which our store is located was sealed by raising barricades, people in the locality began to see our staff and me in a different light” (P-109, 42 years old male)

“Our house has been labelled as CORONA HOUSE in the neighborhood.”(P-29, 42 years old female)

“They sealed the street. The neighbors behaved quite indecently & told the authorities to shove & kick us out of our home. They even accused us of being responsible of spreading the illness in the area.” (P-26, 80 years old male)

### Humiliating and sarcastic behaviour of others

The extent of disease associated stigma can be inferred from the numerous reports of insensitive strategies employed by health administration and police forces to bring patients in hospital. Like

‘It was embarrassing how the administration brought policemen at my workplace after I tested positive for corona.” (P-04, 20years old female)

Police and Health care officers came to my house and blocked the streets, closed the shutters of the shops. I felt like a criminal being taken away. What wrong had I done? (P-111,32 year old male)

Before the Imam who used to lead Friday prayers in our store was on his way to his village when his brother called. He was told that authorities made announcement from village mosque stating that Imam should not be allowed to enter the village as police is there to take him away. He hid in a nearby village and was very distressed” (P-109,42 years old male)

### Breach of confidentiality & loss of trust and respect

During the pandemic, patients’ names and details were posted on social media. This resulted in serious distress and difficult situation for family and friends.

‘Pictures and videos of my family members circulated on social media which revealed our identities even our house address..’ (P-18,50 years old female; P-108,25 years old female)

“My son & I tested positive in the family. When the authorities came to take us away, people in the street were making videos & staring at us strangely. I felt so exposed & humiliated & I feel I can never regain that respect” (P-38, 37 years old female)

Many whipped out their mobile phone cameras to shoot a video of us and also shared status at Facebook, while others simply stared”. (P-111,32 years old male)

“We were told that your tests done were faulty and need to be redone. We were taken to hospital by giving wrong information and after waiting for two hours told that we can’t go home due to us being infected. Why lie to us in the first place; how can I trust anything you people say now”. (P-112,33 years old male)

### Stigma experienced and economic impact

The possible negative impact of COVID-19 on patient business was also a concern.

‘I used to teach kids at home, the neighbors will now be hesitant in sending over their children for tuitions as our house has been labelled as CORONA HOUSE.’ (P-29,42 years old female)

*“This has definitely affected our livelihood as people would boycott our family business*.

However, we will stay in the business”. (P-109,42 years old male)

## DISCUSSION

Our study demonstrates the existence of significant stigma among COVID-19 patients isolated in a tertiary care hospital in a LMIC. To our knowledge, this is the first study from LMIC on this aspect. COVID -19 illness manifests many disease characteristics likely to evoke stigma: the illness being considered by others as the bearer’s *responsibility*, the illness being *fatal* and *contagious* and due to quarantine and isolation becoming quickly *apparent* to others.^12^ COVID-19 positive cases and families, those in isolation or quarantine, whether they have tested positive or not were also identified as one of the groups likely to be discriminated in neighbouring country India.^6^ Patients in our sample scored high on concerns about public attitudes and disclosure concerns. These results are comparable to a study of HIV patients in Sweden: Mean score on concerns about public attitudes were reported as 7.60 (2.50) vs 7.43(1.43) in COVID-19 patients, while disclosure concerns were high in AIDS patients [9.08 (2.57) vs 6.9(1.45)].^13^ The extent of COVID-19 associated stigma can be inferred from numerous experiences patients narrated of verbal abuse, social ostracization of patients and their families and communities in our sample as well as recent literature.^6^ These concerns of COVID-19 patients may increase the psychological suffering of individual patients and may also delay the medical help seeking resulting in poorer medical outcomes. In addition, the same stigma can worsen community outcomes of the illness spread since stigmatized patients and families, not tested and quarantined appropriately can continue to spread the disease widely in the community.

A sense of fear has surrounded COVID-19 since the beginning of this pandemic. There are concerns that compared to other infectious disease epidemics, fear and stigma is perhaps more intense during this pandemic.^14^ Various reasons have been highlighted in the literature for this observation including the novel nature of the infectious agent and the disease, lack of reliable information and unknown risks of infection especially during the early days of the pandemic.^6^ The negative media coverage including the use of scientifically misleading and stigmatizing phrases such as “Chinese Virus”^15^ and the rapid global spread of fake news and false information termed “infodemic” by the WHO^16^ are also contributing to the stigma of disease. Unfortunately in countries like Pakistan, the government policies of enforcing forced lockdown in the country and law enforcement practices for combatting the disease further fuel fear and stigma.^6^,^10^ Stigma is likely being perpetuated on social media platforms like Twitter as well.^5^

Available evidence suggests that in male dominated society like Pakistan, women are more likely to suffer from stigma than their male counterparts.^17^ However, our study showed the opposite. Male patients had statistically significant “personalized stigma” in our sample which is described as “perceived consequences of other people knowing that the respondent has COVID-19, such as losing friends, feeling that people were avoiding him/her, and regrets for having told people.^11^ Men overall showed higher mean scores in other stigma domains too (Table-III). Further research is needed to explore the reasons for these findings. Stigma and its psychological impact have been a major theme in the literature on infectious diseases outbreaks like Plague, Cholera, Influenza, SARS, and AIDS etc.^18^,^19^,^20^ As rightly pointed out by Banerjee and Bhattacharya (2020) in a recent editorial “Pandemics are not just medical phenomena. They have immense psycho-social implications, affecting society at large”.^21^

Our findings have serious public health implications. At the individual level it may be linked with postponing or rejecting care, non-adherence to restrictive measures and treatment, physical and psychological stress and self-stigma. The spate of suicides linked to COVID-19 globally highlight the self-stigma.^22^,^23^ Stigma is also linked with problems in detection and control of the disease, prejudice against whole communities and increased mortality and morbidity leading to negative impacts on public health and pandemic response. The persistent stigma and false beliefs associated with the disease may become a major hurdle against effective public health interventions such as vaccination, as has been the case in polio vaccination programme in Pakistan. Mak (2006) rightly pointed out that ‘*for preventive programs of infectious diseases to be effective, their associated stigma must be actively addressed”*.^24^ Addressing stigma and discrimination towards individuals affected by COVID-19 should therefore be a priority for public health professionals.

Combatting stigma requires that culturally sensitive evidence-based strategies to reduce the stigma of COVID-19 are developed. Lessons learned from the successful experience of stigma-addressing measures in past epidemics and the guidelines developed by the WHO to address stigma^25^ can help to inform the public health campaigns to fight the stigma. These public health interventions to address the COVID-19 related stigma must impart clear, complete and authentic information regarding illness and rationale for disease containment measures through multiple, culturally appropriate channels including town hall meetings, places of worships, and health education channels to complement mass media messages.

A recent initiative at our institution where all confirmed or suspected COVID-19 hospitalized patients and their families were offered patient centered mental health services and education to impart the education regarding the nature of illness and disease containment measures can be a useful model for LMIC settings. All admitted patients received telephonic counselling, were provided the telephone numbers of the newly established telepsychiatry service and were encouraged to seek counselling at any time while hospitalized. Medical teams looking after COVID-19 patients were provided online Psychological First Aid (PFA) training aimed at giving them skills to alleviate symptoms of distress in patients

### Limitation of the study

The limitations of study include a sample consisting of only hospitalized patients in one tertiary care hospital setting. The Stigma scale was adopted from an HIV AIDS scale, which was not validated for the COVID-19 related stigma. Interviews were conducted by phone and there was a lack of face to face communication due to infection concerns. Despite these limitations, our study has several strengths including detailed in-depth interviews with mixed method approach and patient centered service provision.

## CONCLUSION

To conclude, this study brings attention to the insidious impact of stigma in COVID-19 which can have far reaching consequences. Our study offers important preliminary insights into how the stigma of COVID-19 pandemic is affecting the lives of people in a developing country. Stigma appears to have a corrosive influence on participants’ mental health, in large part through stress and disruption of social support systems and social relationships. It can negatively affect recovery from the illness and also contribute to difficulties in pandemic containment. This stigma may persist over time as observed in the SARS and AIDS outbreaks. Multicentre studies, both prospective and retrospective, with larger sample sizes are needed to understand the long- term mental health consequences of this devastating global pandemic.

### Authors’ contributions:

**NI:** Conceived the idea of this study. **NI, IA, HA:** Collected and analyzed data, prepared tables, and wrote the first draft of manuscript. **AH, BS, AA & SF:** Helped with Writing-Reviewing and Editing. **NI:** Was responsible for the supervision of this project. All authors approved the final version of this review article.
